# A Complete Genome Screening Program of Clinical Methicillin-Resistant Staphylococcus aureus Isolates Identifies the Origin and Progression of a Neonatal Intensive Care Unit Outbreak

**DOI:** 10.1128/JCM.01261-19

**Published:** 2019-11-22

**Authors:** Mitchell J. Sullivan, Deena R. Altman, Kieran I. Chacko, Brianne Ciferri, Elizabeth Webster, Theodore R. Pak, Gintaras Deikus, Martha Lewis-Sandari, Zenab Khan, Colleen Beckford, Angela Rendo, Flora Samaroo, Robert Sebra, Ramona Karam-Howlin, Tanis Dingle, Camille Hamula, Ali Bashir, Eric Schadt, Gopi Patel, Frances Wallach, Andrew Kasarskis, Kathleen Gibbs, Harm van Bakel

**Affiliations:** a Department of Genetics and Genomic Sciences, Icahn School of Medicine at Mount Sinai, New York City, New York, USA; b Icahn Institute for Data Science and Genomic Technology, Icahn School of Medicine at Mount Sinai, New York City, New York, USA; c Department of Medicine, Division of Infectious Diseases, Icahn School of Medicine at Mount Sinai, New York City, New York, USA; d Department of Pathology, Clinical Microbiology, Icahn School of Medicine at Mount Sinai, New York City, New York, USA; e Department of Medicine, Division of Infectious Diseases, Northwell Long Island Jewish Medical Center, New Hyde Park, New York, USA; f Division of Neonatology and Department of Pediatrics, The Children’s Hospital of Philadelphia, The University of Pennsylvania, Philadelphia, Pennsylvania, USA; National Institute of Allergy and Infectious Diseases

**Keywords:** MRSA, NICU outbreak, genome analysis

## Abstract

Whole-genome sequencing (WGS) of Staphylococcus aureus is increasingly used as part of infection prevention practices. In this study, we established a long-read technology-based WGS screening program of all first-episode methicillin-resistant Staphylococcus aureus (MRSA) blood infections at a major urban hospital. A survey of 132 MRSA genomes assembled from long reads enabled detailed characterization of an outbreak lasting several months of a CC5/ST105/USA100 clone among 18 infants in a neonatal intensive care unit (NICU). Available hospital-wide genome surveillance data traced the origins of the outbreak to three patients admitted to adult wards during a 4-month period preceding the NICU outbreak. The pattern of changes among complete outbreak genomes provided full spatiotemporal resolution of its progression, which was characterized by multiple subtransmissions and likely precipitated by equipment sharing between adults and infants. Compared to other hospital strains, the outbreak strain carried distinct mutations and accessory genetic elements that impacted genes with roles in metabolism, resistance, and persistence. This included a DNA recognition domain recombination in the *hsdS* gene of a type I restriction modification system that altered DNA methylation. Transcriptome sequencing (RNA-Seq) profiling showed that the (epi)genetic changes in the outbreak clone attenuated *agr* gene expression and upregulated genes involved in stress response and biofilm formation. Overall, our findings demonstrate the utility of long-read sequencing for hospital surveillance and for characterizing accessory genomic elements that may impact MRSA virulence and persistence.

## Abstract (precis)

Whole-genome sequencing (WGS) of Staphylococcus aureus is increasingly used as part of infection prevention practices. In this study, we established a long-read technology-based WGS screening program of all first-episode methicillin-resistant Staphylococcus aureus (MRSA) blood infections at a major urban hospital.

## INTRODUCTION

Health care-associated infections (HAI) with methicillin-resistant Staphylococcus aureus (MRSA) are common, impair patient outcomes, and increase health care costs (1, 2). MRSA is highly clonal, and much of our understanding of its dissemination has relied on lower-resolution molecular strain typing methods such as pulsed-field gel electrophoresis (PFGE), S. aureus protein A (*spa*) typing, and multilocus sequence typing (MLST) (3) and typically includes characterization of accessory genome elements that define certain lineages and are implicated in their virulence. Examples of the latter include the arginine catabolic mobile element (ACME), S. aureus pathogenicity island 5 (SaPI5), and the Panton-Valentine leukocidin (PVL)-carrying ϕSa2 prophage in the community-associated (CA) CC8/USA300 lineage (4, 5). Molecular typing facilitates rapid screening but has limited resolution to identify transmissions within clonal lineages. Moreover, genetic changes can lead to alteration or loss of typing elements (6–9). As such, whole-genome sequencing (WGS) has emerged as the gold standard for studying lineage evolution and nosocomial outbreaks (10, 11). Transmission analysis with WGS has been performed largely retrospectively to date (12–15), although prospective screening with resulting interventions has also been described (10, 16).

In addition to lineage and outbreak analysis, WGS has furthered our understanding of S. aureus pathogenicity by delineating virulence and drug resistance determinants (17, 18), including those related to adaptation to the hospital environment (17, 19). Many of these elements are found in nonconserved “accessory” genome elements that include endogenous prophages, mobile genetic elements (MGEs), and plasmids (20, 21). The repetitive nature of many of these elements means that they are often fragmented and/or incompletely represented in most WGS studies to date due to limitations of commonly used short-read sequencing technologies, curbing insights into their evolution (21). Recent advances in throughput of long-read sequencing technologies now enable routine assembly of complete genomes (22, 23) and analysis of core and accessory genome elements (13, 18), including DNA methylation patterns (24), but these technologies have not yet been widely used for prospective MRSA surveillance.

Here, we describe the results of a complete genome-based screening program of MRSA blood isolates. During a 16-month period, we obtained finished-quality genomes for first blood isolates from all bacteremic patients. In addition to providing detailed contemporary insights into prevailing lineages and genome characteristics, we characterized widespread variation across accessory genome elements, impacting loci encoding virulence and resistance factors, including those commonly used as molecular strain typing markers. We also found multiple transmission events not recognized based on epidemiological information. During an outbreak event in the neonatal intensive care unit (NICU), our program was able to provide actionable information that discriminated outbreak-related transmissions, identified individual subtransmission events, and traced the NICU outbreak origin to adult hospital wards. Finally, comparative genome and gene expression analyses of the outbreak clone to hospital background strains identified genetic and epigenetic changes, including acquisition of accessory genome elements, which may have contributed to the persistence of the outbreak clone.

## MATERIALS AND METHODS

### Ethics statement.

This study was reviewed and approved by the Institutional Review Board of the Icahn School of Medicine at Mount Sinai and the MSH Pediatric Quality Improvement Committee at Mount Sinai Hospital.

### Case review.

An investigation of the characteristics of the patients included review of existing medical records for relevant clinical data. Unique ventilator identification numbers and the real-time location system (RTLS) enabled mapping of ventilator locations over time.

### Bacterial isolate identification and susceptibility testing.

Isolates were grown and identified as part of standard clinical testing procedures in the Mount Sinai Hospital Clinical Microbiology Laboratory (CML, and stored in tryptic soy broth (TSB) with 15% glycerol at −80°C. Species confirmation was performed with matrix-assisted laser desorption ionization–time of flight (MALDI-TOF) (Bruker Biotyper; Bruker Daltonics). Vitek 2 (bioMérieux) automated broth microdilution antibiotic susceptibility profiles were obtained for each isolate according to Clinical and Laboratory Standards Institute (CLSI) 2015 guidelines and reported according to CLSI guidelines (25). Susceptibility to mupirocin was determined by Etest (bioMérieux), and susceptibility to chlorhexidine was tested with discs (Hardy) impregnated with 5 μl of a 20% chlorhexidine gluconate solution (Sigma-Aldrich).

### DNA preparation and sequencing.

For each isolate, single colonies were selected and grown separately on tryptic soy agar (TSA) plates with 5% sheep blood (blood agar) (Thermo Fisher Scientific) under nonselective conditions. After growth overnight, cells underwent high-molecular-weight DNA extraction using the Qiagen DNeasy Blood & Tissue kit (Qiagen) according to the manufacturer’s instructions, with modified lysis conditions: bacterial cells were pelleted and resuspended in 300 μl enzymatic lysis buffer (20 mM Tris-Cl [pH 8.0], 2 mM sodium EDTA), 3 μl of 100 mg/ml RNase A (AM2286; Ambion), and 10 μl of 100 mg/ml lysozyme (L1667-1G; Sigma) for 30 min at 37°C, followed by the addition of 25 μl proteinase K (20 mg/ml) (Qiagen) and 300 μl Buffer AL and further incubation for 1 h at 56°C, after which two rounds of bead beating (BioSpec) were performed of 1 min each using 0.1-mm silica beads (MP Bio) (13).

Quality control, DNA quantification, library preparation, and sequencing were performed as described previously (13). Briefly, DNA was gently sheared using Covaris G-tube spin columns into ∼20,000-bp fragments and end-repaired before ligating SMRTbell adapters (Pacific Biosciences). The resulting library was treated with an exonuclease cocktail to remove unligated DNA fragments, followed by two additional purification steps with AMPure XP beads (Beckman Coulter) and Blue Pippin (Sage Science) size selection to deplete SMRTbells of <7,000 bp. Libraries were then sequenced using P5 enzyme chemistry on the Pacific Biosciences RS-II platform to >200× genome-wide coverage.

### Complete genome assembly and finishing.

PacBio SMRT sequencing data were assembled using a custom genome assembly and finishing pipeline (26). Briefly, sequencing data were first assembled with HGAP3 version 2.2.0 (22). Contigs with less than 10× coverage and small contigs that were completely encompassed in larger contigs were removed. Remaining contigs were circularized and reoriented to the origin of replication (*ori*) using Circlator (27) and aligned to the nonredundant nucleotide collection using BLAST+ (28) to identify plasmid sequences. In cases where chromosomes or plasmids did not assemble into complete circularized contigs, manual curation was performed using Contiguity (29). Genes were annotated using PROKKA (30) and visualized using ChromoZoom (31) and the Integrated Genome Browser (IGB) (32). Interproscan (33) was used to annotate protein domains and Gene Ontology (GO) categories for annotated genes.

### Resolution of large genomic inversions.

To resolve inversion events catalyzed by two prophage elements (*Staphylococcus* ϕSa1 and Staphylococcus aureus ϕSa5) with large (>40 kbp) nearly identical regions present in some of the assembled genomes, we developed a phasing approach that took advantage of unique variants present in each element (see Fig. S1 in the supplemental material). Raw (i.e., uncorrected) PacBio reads were first mapped to one of the repeat copies using BWA-MEM (34). Variants were then called with Freebayes (35), and high-quality single nucleotide variants with two distinct alleles of approximately equal reads coverage were identified. Analogous to procedures used in haplotype phasing, we then determined which variant alleles were colocated in the same repeat element: if at least three-quarters of the raw reads containing a particular allele also encompassed distinct allele(s) of a neighboring variant(s), the alleles were considered linked. In all cases, this resulted in two distinct paths through the repeated prophage elements that were each linked to a unique sequence flanking each repeat. We then used this information to correct assembly errors and identify bona fide inversion events between isolate genomes. Final verification of corrected assembly was performed by examining the phasing of the raw reads with HaploFlow (36).

### Phylogenetic reconstruction and molecular typing.

Phylogenetic analyses were based on whole-genome alignments with parsnp (37), using the filter for recombination. The VCF file of all variants identified by parsnp was then used to determine pairwise single nucleotide variant (SNV) distances between the core genomes of all strains. For visualization of the whole-genome alignments, isolate genomes were aligned using sibelia (38) and processed by ChromatiBlock (https://github.com/mjsull/chromatiblock). Indels, structural variants, and SNVs in pairwise complete genome alignments were identified using NucDiff (39).

The multilocus sequence type was determined from whole-genome sequences using the RESTful interface to the PubMLST S. aureus database (40). Staphylococcal protein A (*spa*) typing was performed using a custom script (https://github.com/mjsull/spa_typing). SCC*mec* typing was done using SCC*mec*Finder (41). Changes to ACME and SaPI5 were determined using BLASTn and Easyfig. Presence or absence of genes in each locus was determined using BLASTx (42), and a gene was considered to be present if 90% of the reference sequence was aligned with at least 90% identity. Prophage regions were detected using PHASTER. Each region was then aligned to a manually curated database of S. aureus phage integrases using BLASTx to identify their integrase group.

### Annotation of antibiotic resistance determinants.

Antibiotic resistance genes and variants were annotated by comparing to a manually curated database of 39 known S. aureus resistance determinants for 17 antibiotics compiled from the literature. BLAST (42) was used to identify the presence of genes in each isolate genome, with a sequence identity cutoff of ≥90% and an E value cutoff of ≤1e−10. Resistance variants were identified by BLAST alignment to the reference sequence of the antibiotic resistance determinant. Only exact matches to variants identified in literature were considered.

### Identification of NICU outbreak subgroups.

Changes between each outbreak isolate and the p133 reference isolate were identified using GWviz (https://github.com/mjsull/GWviz), which uses nucdiff (39) to identify all genomic variants between pairs of strains and then uses PROKKA gene annotations to determine the effect of the change on coding regions. nucdiff in turn uses nucmer to find alignments between two genomes and then identifies large structural rearrangements by looking at the organization of nucmer alignments and smaller changes such as SNVs or indels by finding differences between the aligned regions. To confirm indel changes between isolates in the outbreak, small indels were filtered based on underlying read data. Briefly, raw PacBio reads were aligned back to each outbreak genome assembly using BWA-MEM (34). GWviz was then used to determine the number and proportion of raw reads supporting variants in each strain. Variants were selected for further delineation of outbreak subgroups if they were present in two or more isolate genomes and supported by at least ten raw reads in each genome, of which at least 75% confirmed the variant.

A graph of SNV distances between isolates was obtained from a multiple alignment of all outbreak isolates. The minimum spanning tree was then constructed using the minimum spanning tree functionality in the Python library networkX (https://networkx.github.io/).

### Identification of genetic variants unique to the NICU outbreak clone.

To determine SNVs unique to the outbreak isolate the marginal ancestral states of the ST105 isolates were determined using RAxML (43) from a multiple alignment of all ST105s generated using parsnp. We identified all SNVs that had accumulated from the most recent common ancestor of the outbreak strain and the closest related nonoutbreak ST105, and the most recent common ancestor (MRCA) of all outbreak strains. SNVs causing nonsynonymous mutations or changes to the promoter region of a gene (defined as <500 bp upstream of the start site) were plotted. Orthology was assigned using BLASTkoala (44).

Core and accessory gene content in ST105 outbreak and nonoutbreak strains was determined using ROARY. Genes found in more than two outbreak strains and less than 33% of the other ST105 genomes were then plotted along with select methylation data. Phylogenetic reconstruction of ST105 was performed using parsnp, and the resulting tree and gene presence information was visualized using m.viridis.py (https://github.com/mjsull/m.viridis) which uses the python ETE toolkit (45).

### DNA methylation profiling.

SMRT raw reads were mapped to the assembled genomes and processed using smrtanalysis v5.0 (Pacific Biosciences, Menlo Park, CA). Interpulse durations (IPDs) were measured and processed as previously described (24, 46) to detect modified N^6^-methyladenine (m6A) nucleotides.

### RNA preparation and sequencing.

For RNA extraction, overnight cultures in tryptic soy broth (TSB) were diluted (optical density at 600 nm [OD_600_] of 0.05), grown to late-log phase (OD_600_ of ∼0.80) in TSB, and stabilized in RNALater (Thermo Fisher). Total RNA was isolated and purified using the RNeasy Mini kit (Qiagen) according to the manufacturer’s instructions, except that two cycles of 2-min bead beating with 1 ml of 0.1-mm silica beads in a mini bead beater (BioSpec) were used to disrupt cell walls. Isolated RNA was treated with 1 μl (1 unit) of Baseline Zero DNase (Epicentre) at 37°C for 30 min, followed by rRNA depletion using the Epicenter Ribo-Zero Magnetic Gold kit (Illumina), according to the manufacturer’s instructions.

RNA quality and quantity were assessed using the Agilent Bioanalyzer and Qubit RNA Broad Range assay kit (Thermo Fisher), respectively. Barcoded directional RNA sequencing libraries were prepared using the TruSeq Stranded Total RNA Sample Preparation kit (Illumina). Libraries were pooled and sequenced on the Illumina HiSeq platform in a 100-bp single-end read run format with six samples per lane.

### Differential gene expression analysis.

Raw reads were first trimmed by removing Illumina adapter sequences from 3′ ends using cutadapt (47) with a minimum match of 32 bp and allowing for 15% error rate. Trimmed reads were mapped to the reference genome using Bowtie2 (48), and htseq-count (49) was used to produce strand-specific transcript count summaries. Read counts were then combined into a numeric matrix and used as input for differential gene expression analysis with the Bioconductor EdgeR package (50). Normalization factors were computed on the data matrix using the weighted trimmed mean of M values (TMM) method (51). Data were fitted to a design matrix containing all sample groups, and pairwise comparisons were performed between the groups of interest. *P* values were corrected for multiple testing using the Benjamin-Hochberg (BH) method and used to select genes with significant expression differences (*q* < 0.05).

### Data availability.

All genome data and assemblies are available in GenBank under accession numbers CP030375 to CP030714, QNXD00000000, QNXE00000000, QNXF00000000, QNXG00000000, QNXH00000000, QNXI00000000, QNXJ00000000, QNXK00000000, QNXL00000000, QNXM00000000, and VZDK00000000 (see Table S1).

## RESULTS

### Complete genome surveillance reveals accessory genome variation among clonal MRSA lineages.

**FIG 1:**
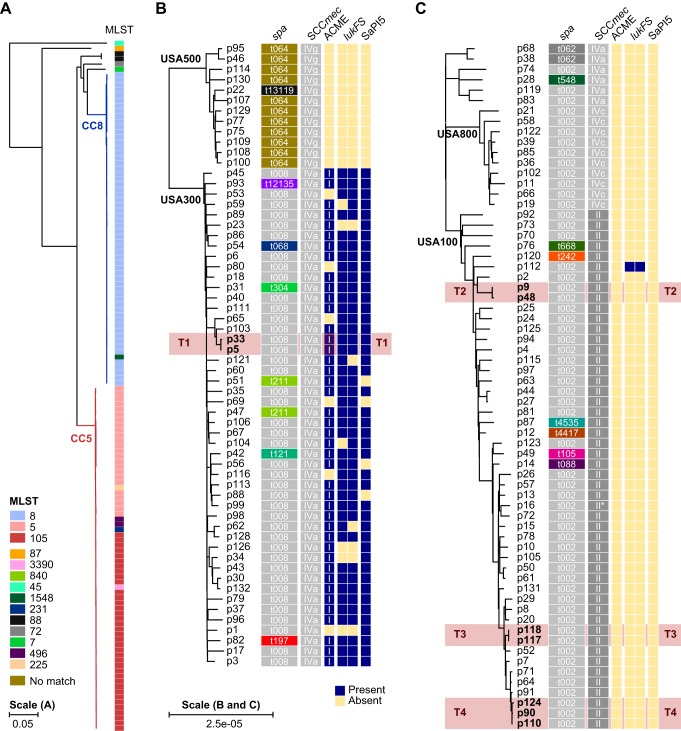
Phylogeny of MRSA bacteremia surveillance isolates. (A) Maximum likelihood phylogenetic tree based on SNV distances in core genome alignments of 132 primary MRSA bacteremia isolates. CC8 and CC5 clades are shaded in red and blue, respectively. Multilocus sequence types (MLST) for each branch are shown as colored blocks, with a key at the bottom left. (B) Enlarged version of the CC8 clade from panel A. The isolate identifier is indicated next to each branch, together with blocks denoting the *spa* type, SCC*mec* type, and the presence (blue) or absence (yellow) of intact ACME, *lukFS*, and SaPI5 loci. The ACME type is indicated in each box. The *lukFS* locus is represented by two blocks indicating the presence of *lukF* and *lukS*, respectively. (C) Same as panel B, but for the CC5 clade. *, *spa* type II isolate with an inserted element in the locus. Four transmission events between patients are highlighted in red and labeled T1 to T4. Scale bars indicate the number of substitutions per site in the phylogeny.

To characterize the genetic diversity of MRSA blood infections at The Mount Sinai Hospital (MSH) in New York City, NY, we sequenced the first positive isolate from all 132 MSH inpatients diagnosed with MRSA bacteremia between fall 2014 and winter 2015. Single-molecule real-time (SMRT) long-read length RS-II WGS was used to obtain finished-quality chromosomes for 122 of 132 isolates (92%), along with 145 unique plasmids across isolates (see Table S1 in the supplemental material). The remaining isolates had one or more chromosomal contigs that could not be closed with available long-read sequencing data. We reconstructed a phylogeny from a multigenome alignment (Fig. 1A; see also Fig. S2A), which identified two major clades corresponding to S. aureus clonal complexes 8 (CC8; 45.5% of isolates) and 5 (CC5; 50% of isolates) based on the prevailing multilocus sequence types (STs) in each clade (ST8 and ST105/ST5, respectively). The CC8 isolates further partitioned among the endemic community-associated (CA) USA300 (80%) and the hospital-associated (HA) USA500 (20%) lineages (Fig. 1B), while CC5 isolates mainly consisted of USA100 (75.8%) and USA800 (15.2%) HA lineages (Fig. 1C). Overall, the phylogeny was consistent with the major MRSA lineages found in New York City, NY, and the United States (52).

We further examined larger (>500 bp) structural variation that may be missed by short-read-based WGS approaches (13, 14, 53). The multigenome alignment indicated that between 80.8% and 88.9% of the sequence in each genome was contained in core syntenic blocks shared among all 132 genomes see (Fig. S2A). Another 9.5% to 16.8% was contained in accessory blocks found in at least two but not all genomes. Many of these accessory genome elements were lineage specific and associated with prophage regions and plasmids (Fig. S2B). Finally, 0.8% to 4.5% of the sequence was not found in syntenic blocks and included unique elements gained by individual isolates. The extent of core and accessory genome variability impacted loci that are commonly used for molecular strain typing. Divergence from the dominant *spa* type was apparent in 8 (13.3%) of CC8 and 9 (13.6%) of CC5 lineage isolates. MLST loci were more stable in comparison with changes in 1.5% and 7.6% of isolates in each lineage, respectively. Notably, there were also widespread changes at ACME, PVL, and SaPI5 (Fig. 1B) in USA300 isolates, which are signature elements of this CA lineage (4, 5); 33.3% (16 of 48) either carried inactivating mutations or had partially or completely lost one or more elements (Fig. 1B). The multiple independent events of ACME, PVL, and SaPI5 loss throughout the USA300 clade may reflect its ongoing adaptation to hospital environments, as these elements are typically absent in HA lineages. Interestingly, we found one case of a PVL-positive USA100 isolate (Fig. 1C) that may have resulted from homologous recombination between a ϕSa2 and ϕSa2 PVL prophage (see Fig. S3). Thus, complete genomes of MRSA blood isolates demonstrate the mobility of the accessory genome in ways that impact commonly used S. aureus lineage definitions.

### Identification of transmission events among adults and an outbreak in the NICU.

We next compared isolate genomes to identify transmissions between patients. To establish similarity thresholds for complete genomes obtained from long-read SMRT sequencing data, considering both intrahost diversity and genetic drift, we first examined baseline single nucleotide variant (SNV) distances within each lineage. Median pairwise genome differences ranged from 101 SNVs for USA800 to 284 SNVs for USA100 (see Fig. S4A). We also examined the extent of divergence among 30 bacteremia isolate pairs collected within a span of 1 month to 1.4 years from individual patients. Pairwise distances for within-patient isolates were substantially lower than the median for each lineage (Fig. S4B to E), consistent with persistent carriage of the same clone (54, 55), with no more than 10 SNVs separating isolate pairs. Small (<5 bp) indels were more common than SNVs and mostly associated with homopolymer regions that can be problematic to resolve with third-generation sequencing technologies, indicating that they likely reflected sequencing errors. Notably, several patients showed variation between isolates collected within a span of several days (Fig. S4B to E), indicative of intrahost genetic diversity. As such, we considered intrahost diversity and genetic drift in aggregate and set a conservative distance of ≤7 SNVs to define transmission events in our genome phylogeny. At this threshold, we identified one USA300 and three USA100 transmissions involving six adults and three infants (Fig. 1B and C, labeled T1 to T4). Complete pairwise genome alignments for each event confirmed the absence of structural variants. In the USA300 transmission case (T1), the presumed index patient p5 was bacteremic with the same clone on two occasions ∼3 months apart (see Fig. S5). The recipient (p33) was admitted to the same ward and overlapped with p5 for 7 days prior to the time of bacteremia. The USA100 isolates in transmission T2 were collected ∼4 months apart, and although the patients had overlapping stays, they did not share a ward or other clear epidemiological links (Fig. S5). In transmission T3, patients shared a ward for several days (Fig. S5).

The final transmission involving 3 infants (T4) was part of a larger outbreak in the NICU, where positive clinical MRSA cultures from three infants within 5 weeks had prompted an investigation and consultation with the New York State Department of Health (NYSDOH). During 4 months, an additional 41 clinical and surveillance cultures from 20 infants tested positive for MRSA, bringing the total to 46 isolates from 22 infants. Three further isolates were obtained from incubators and an intravenous (i.v.) box, from a total of 123 environmental swabs (2.4%). Positive nasal surveillance cultures were also obtained from 2 of 130 (1.5%) health care workers (HCWs) who had provided direct care to newly MRSA-colonized infants. The NYSDOH performed PFGE on 22 isolates, of which 14 patients and 3 environmental isolates had nearly indistinguishable band patterns (data not shown). This included p90 and p110 in transmission T4 (Fig. 1C) (p125 was not tested). The USA100 (ST105) outbreak clone was resistant to fluoroquinolones, clindamycin, gentamicin, and mupirocin and susceptible to vancomycin, trimethoprim-sulfamethoxazole, and doxycycline (see Fig. S6 and Table S1). This pattern was uncommon (18.2%) among USA100 isolates in our study and was therefore used as an initial screening criteria for cases. None of the HCW isolates matched the MLST or antibiogram of the outbreak clone, and both staff members were successfully decolonized with nasal mupirocin and chlorhexidine gluconate (CHG) baths.

### Complete genome surveillance resolves outbreak origin and progression.

**FIG 2:**
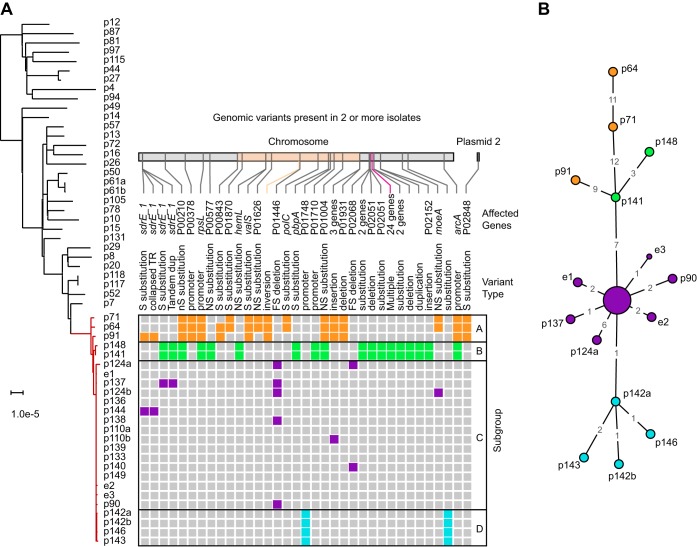
NICU outbreak subgroups and association with adult bacteremia patients. (A) Maximum likelihood phylogenetic tree based on SNV distances in core genome alignments of 31 ST105 primary bacteremia isolates (black) and 25 outbreak isolates (red). The core genome makes up 76.1% to 82.6% of each genome. The scale bar indicates the number of substitutions per site. The patient (p) or environmental (e) isolate identifier is shown next to each branch (a/b suffixes indicate multiple isolates from the same patient). Variants present in two or more NICU outbreak isolates, derived from full-length pairwise alignments to the p133 genome, are shown as colored boxes. Variants are colored according to outbreak subgroups inferred from common variant patterns, as indicated on the right. For each variant the genomic location, affected genes, and type of mutation is shown above the matrix. A 2-Mbp inversion in the adult isolates and a 2,411-bp region containing two substitutions and a deletion in subgroup Bare highlighted in the location bar in orange and purple, respectively. (B) Minimum spanning tree of the 25 outbreak isolates based on SNVs identified in the complete genome alignment of all ST105 isolates. The 15 labeled nodes represent individual isolates. The larger central node corresponds to ten isolates with identical core genomes, which includes the p133 reference. Nodes are colored according to the outbreak subgroups shown in panel A. Numbers at edges represent core genome SNV distances.

During the outbreak, we expanded our genomic screening program to include the first isolate of suspected outbreak cases. From day 354 onwards, we obtained 23 additional complete genomes (Table S1). Of these, 19 genomes from 16 infants and three environmental isolates matched the ST105 outbreak strain type, bringing the total to 22 outbreak genomes from 16 infants and the environment. The infection prevention team and the NYSDOH were informed of isolate genomes meeting our transmission threshold within 10 to 14 days of a positive test, which helped delineate the final case set and determine when the outbreak ended. Further analyses were performed retrospectively to reconstruct the chain of events that initiated and sustained the outbreak. To this end, we first reconstructed a phylogenetic tree based on core genome alignments of all ST105 isolates in our study, which grouped all 22 isolates with matching antibiograms and/or PFGE patterns in one well-defined clade (Fig. 2A). Surprisingly, this clade also contained 3 MRSA isolates obtained from adult bacteremia patients in other hospital wards prior to the first NICU case. The outbreak clade genomes were ≤15 SNVs apart, and the clade as a whole differed from other ST105 isolates by ≥41 SNVs. We therefore considered the 3 adult isolates to be part of a larger clonal outbreak that spanned 7 months. The availability of complete genome sequences provided additional genomic variants that contributed to strain diversity within the outbreak clade, including indels, structural variants, and a large megabase-size inversion (Fig. 2A). Based on these variant patterns, we distinguished 4 distinct subgroups. A minimum spanning tree based only on core genome SNVs that is more commonly used in outbreak investigations using short-read sequencing data largely recapitulated the same grouping (Fig. 2B) but with reduced resolution between subgroups A and B.

**FIG 3:**
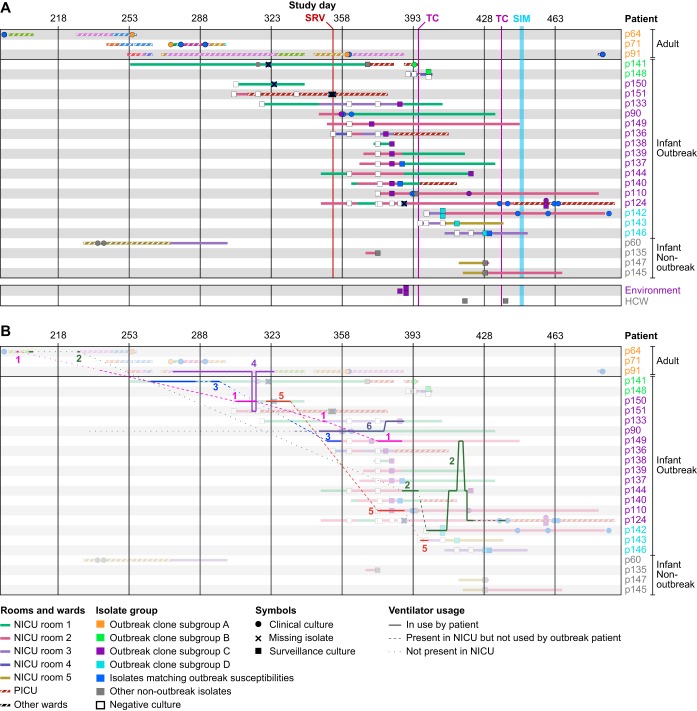
Timeline of the NICU outbreak. (A) Overview of outbreak patient stays and isolates collected during the NICU outbreak. Rows correspond to patients with admission periods shown as horizontal bars. Solid fill patterns denote NICU stays and striped patterns indicate stays in other MSH wards. Fill colors correspond to NICU rooms (solid) or hospital wards (striped). Clinical or surveillance isolates collected during each stay are indicated by symbols, with a key shown below. Patient identifiers and isolate symbols are colored by outbreak subgroup. Timeline scale and key interventions are shown at the top. SRV, start of biweekly surveillance cultures; TC, terminal cleaning; SIM, *in situ* simulation. (B) Same as panel A, but with ventilator movements between patients and locations overlaid as lines. Ventilators are numbered and shown in distinct colors. Solid lines correspond to periods that a ventilator was in use by an outbreak patient. Dashed lines indicate when a ventilator was present in the NICU but not used by an outbreak patient. Dotted lines indicate when a ventilator was not in use by an outbreak patient and not present in the NICU. Background colors are muted to facilitate tracking of ventilator movements.

We then used the available epidemiology and genomic data to reconstruct an outbreak timeline (Fig. 3A). The three initial adult cases had overlapping stays and shared wards, and their isolates clustered together in subgroup A. Several of the earliest clinical isolates from infants p141, p150, and p151 that coincided with the spread of the outbreak to the NICU were not available for genomic analysis (marked X in Fig. 3A). The missing isolate from p141 was susceptible to gentamicin and differed from the PFGE pattern of the outbreak clone by five bands. The other two missing isolates from p150 and 151 matched the outbreak clone antibiogram and were therefore considered to be part of the outbreak. Subsequent cases were identified by positive surveillance cultures on days 357 to 386, and their isolates clustered in subgroup C. All but one of the infants in this subgroup stayed in NICU room 2 before or at the time of culture positivity. The three positive environmental isolates were also obtained from this room, suggesting that a local bioburden led to a high volume of colonized infants in a short time. Construction in the NICU and a resulting disruption of infection prevention practices was believed to play a role in the initial transmissions of MRSA.

The increase in new cases on surveillance prompted a terminal clean (TC) of the NICU on day 395. During this time, all infants were temporarily transferred to two different locations. Infant p148 who was colonized with the outbreak clone was placed across the hall from p141 in the pediatric intensive care unit (PICU). A positive surveillance culture in the same subgroup (B) as p141 was obtained for p148 shortly afterwards (Fig. 3A), suggesting that a transmission had occurred during the TC. New positive surveillance cultures were subsequently found for three additional infants (p142, p143, and p146). Each had been admitted after the TC and stayed in room 3 before or at the time of culture positivity. Their isolates comprised subgroup D, suggesting that the outbreak clone spread to this location from the closely related subgroup C linked to room 2 (Fig. 2B and 3A). Thus, each outbreak subgroup (A to D) was associated with a specific area (adult wards, PICU, and NICU rooms 2 and 3, respectively), indicating that location sharing was a dominant factor in the spread of the outbreak clone.

The continued transmissions after the first TC prompted *in situ* simulation and a second TC (Fig. 3A). The simulation efforts reinforced the importance of compliance to infection prevention strategies, patient cohorting, enhanced environmental disinfection, and limiting patient census to decrease bioburden (56). Only one new case (p124) was detected after the second TC. Infant p124 was located the PICU at the time of detection, and based on the genomic profile (subgroup C) and earlier positive isolates, the transmission was believed to have occurred prior to the final TC and *in situ* simulation. As such, the workflow improvements were effective in halting the outbreak. The weekly surveillance cultures ended after three consecutive weeks of negative cultures (day 452). The last colonized patient was discharged 2 months later, and we did not detect the outbreak clone in our hospital-wide genomic screening program in the subsequent 2 years. While the majority of cases were positive by surveillance, there was morbidity related to the outbreak; five infants developed clinical infections, with three bacteremias, one pneumonia, and one surgical site infection. There were no deaths related to the outbreak.

### Ventilator sharing implicated in the origin and progression of the NICU outbreak.

Location and HCW sharing could not account for the link between adult and pediatric cases, which were housed in different buildings and cared for by different HCWs. We focused on a potential role of ventilators in the outbreak based on the observation that (i) all NICU outbreak cases were on invasive or noninvasive ventilator support prior to culture positivity, (ii) the three adult patients were ventilated for at least part of their hospitalizations, and (iii) prior to identification of the NICU outbreak, ventilators were shared between adult and pediatric wards. Ventilator exchange between units was discontinued after the first NICU cases were identified.

The ventilators that were present in the NICU at the time environmental surveillance was performed tested negative for MRSA, but we could not rule out earlier contamination or contributions of other ventilators. Retrospective analysis of equipment usage logs and tracking data provided by the hospital’s real-time location system (RTLS) identified six units that were shared between outbreak cases (Fig. 3B, numbered 1 to 6). Ventilator 1 was briefly used by adult p64 and then transferred to several locations before it was moved into the NICU and later used by infant p150. The first NICU isolate that matched the outbreak clone by antibiogram was isolated from this patient soon after (Fig. 3A and B). Ventilator 4 was used by adult p91 several weeks before this patient developed bacteremia, except for a 2-day period when it was used by infant p151, shortly before the first NICU outbreak case (Fig. 3B). Infant p151 was in the neighboring PICU at this time and remained there until a positive surveillance isolate was obtained. Finally, ventilator 2 was used by adult p64 in two separate hospital visits, but was only moved to the NICU after the outbreak had already spread there.

Within the NICU, the sequential use of ventilator 6 by patients p90 and p133, the timing of their respective culture positivity, and the similarity of their isolate genomes all supported a role for this ventilator in the transmission to p133. Likewise, ventilators 2 and/or 5 may have been a factor in the spread from room 2 (subgroup C) to room 3 (subgroup D), especially considering that both rooms were cleaned just prior to the transmission (Fig. 3A). Ventilator 5 may also have been a transmission vector from p150 to p110. Ventilator 3 was used by p141 and later by p149; however, it is unclear if it played a role in the outbreak, as the first two isolates obtained from p141 after ventilator 3 exposure did not match the outbreak. Altogether, the epidemiological and genomic data suggest that ventilators not only played a role in spreading the outbreak from adult wards to the NICU but were also a factor in subsequent subtransmissions within the NICU.

### Mutations in the outbreak clone alter expression of virulence and persistence factors.

**FIG 4:**
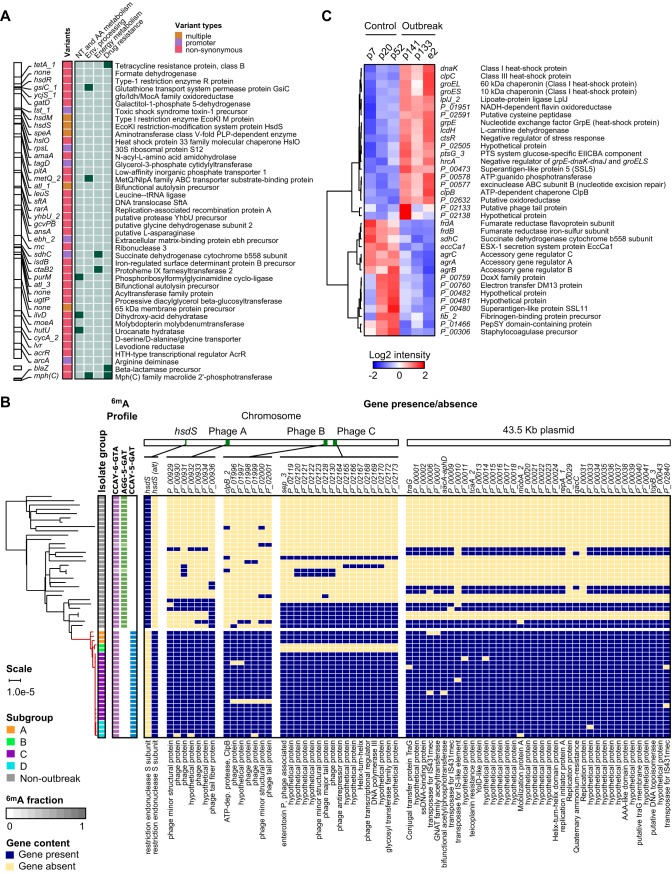
Differentiating features of the NICU outbreak clone compared to the USA100 hospital background. (A) Map of nonsynonymous SNVs in genes and promoter regions that are unique to the outbreak clone. Gene identifiers or names are shown next to their genomic location. The SNV type is indicated by colors with a key shown at the top right. KEGG pathways with two or more genes are indicated on the right (green boxes) and corresponding gene descriptions on the far right. (B) Pan-genome analysis of MLST105 isolates showing all genes present in the outbreak clone and absent from at least half of the nonoutbreak isolates collected during our study. A maximum likelihood phylogenetic tree based on SNV distances in core genome alignments is shown on the left with patient (p) or environmental (e) isolate identifiers. Changes in the m6A methylation profile due to the *hsdS* recombination in the outbreak strain are highlighted in green/blue. Gene presence (yellow) or absence (red) is indicated in a matrix organized by genomic location (top). Gene names and descriptions are shown at the top and bottom of the matrix, respectively. See key on bottom left for more details. (C) Hierarchical clustering of 35 genes with significant expression differences (false-discovery rate [FDR] *q* < 0.05) between three control and three outbreak strains. Columns correspond to control or outbreak isolates, with labels at the top. Gene names and descriptions are shown on the right. Color shades and intensity represent the difference in normalized log2 counts per million (CPM) relative to the average gene expression level, with a color key shown below.

Given the extended duration of the outbreak, we next sought to identify genomic features that could have contributed to its persistence. A comparison of complete genomes found 42 nonsynonymous or deleterious SNVs and indels in the outbreak clone that were not present in any of the ST105 hospital background strains, affecting 35 genes or their promoter regions (Fig. 4A). The products of these genes were primarily involved in nucleotide, amino acid, and energy metabolism as well as environmental signal processing and drug resistance. Several genes encoding cell wall proteins were also affected, including *gatD*, which is involved in amidation of peptidoglycan (57). Pan-genome analysis with Roary (58) further revealed 71 genes exclusive to the outbreak strain or infrequently (<33%) present in other MLST105 isolates (Fig. 4B). Most of these genes were associated with three prophage regions and a 43.5-kbp plasmid. The additional genes in prophage A encoded only phage replication or hypothetical proteins. Among the genes in prophage B was an extra copy of *clpB*, which promotes stress tolerance, intracellular replication, and biofilm formation (59). Prophage C included an extra copy of the *sep* gene encoding an enterotoxin P-like protein associated with an increased risk of MRSA bacteremia in colonized patients (60). The 43.5-kbp plasmid contained the mupirocin (*mupA*) and gentamicin (*aacA-aphD*) resistance genes (see Fig. S6B) that explained the distinct susceptibility profile of the outbreak clone. High-level mupirocin resistance (HLR) conferred by *mupA* has been linked to transmissions in previous studies (61, 62). Pan-genome analysis also revealed a unique variant of the *hsdS* gene in the outbreak strain, which encodes the specificity subunit of a type I restriction modification (RM) system. S. aureus typically contains two type I RM systems that vary in sequence specificity based on the configuration of two target recognition domains (TRDs), which have been categorized according to the DNA motifs they recognize (63). Closer examination revealed that a recombination event in one of the two *hsdS* gene copies present in USA100 (see Fig. S7) changed its TRDs from the typical CC5 “B-D” configuration (recognizing the “AGG-5-GAT” motif present at 738 sites, overlapping 595 genes and 120 promoter regions) to an “A-D” configuration (recognizing the CCAY-5-GAT motif present at 304 sites, overlapping 287 genes and 15 promoter regions), resulting in altered genome-wide m6A DNA methylation profiles compared to those of other ST105 isolates (Fig. 4B).

We reasoned that the (epi)genetic changes in the outbreak clone could alter gene expression patterns and provide further insights into the effects of these changes. We therefore compared the gene expression profiles of three representative outbreak isolates (i.e., cases) to the three most similar nonoutbreak ST105 strains (i.e., controls) during late-log-phase growth. The control strains shared the 43.5-kbp plasmid and most of the prophage elements with the outbreak strain and demonstrated similar growth characteristics (see Fig. S8). Differential gene expression analysis showed altered expression of 35 genes (Fig. 4C). Two of these genes were mutated in the outbreak clone: a SNP in the promoter region of *sdhC* and a duplication of *clpB*. Methylation changes were found in six genes (17.1%), which was lower than the rate of 27.3% across all genes. Thus, most expression changes appear to be indirect results of (epi)genetic changes. Multiple upregulated genes in the outbreak clone encoded proteins involved in stress and heat shock responses. This included *clpB*, which was increased in copy number in the outbreak versus control strains, but also *dnaK* and *clpC*, which have been linked to biofilm formation in S. aureus and adherence to eukaryotic cells (64, 65). Expression of the gene encoding staphylococcal superantigen-like protein 5 (SSL5) was also increased. SSL5 is known to inhibit leukocyte activation by chemokines and anaphylatoxins (66). Among the downregulated genes, the *agrABC* genes of the accessory gene regulator (*agr*) locus stood out. *agr* is the major virulence regulator in S. aureus (67), and decreased *agr* function in clinical isolates is associated with attenuated virulence and increased biofilm and surface protein expression (68).

## DISCUSSION

In this study, we implemented a complete genome screening program at a large quaternary urban medical center, with the aim of tracking circulating clones, to identify transmission events and to understand the genomic epidemiology of endemic strains impacting human health. The availability of complete genomes allowed us to precisely map all genetic changes between strains, highlighting the presence of substantial structural variation in lineages that are considered highly clonal. The extent of variation due to recombinations in prophages, mobilization of genetic elements, and large genomic inversions also impacted classical *spa*, MLST, and signature virulence and resistance elements used in S. aureus molecular typing schemes. As such, the stability of these elements should be considered when using such schemes for lineage analysis. Complete reconstruction of outbreak genomes provided comprehensive variation data to map subtransmission events during a NICU MRSA outbreak. Finally, the combination of genetic and gene expression differences between the NICU outbreak clone and USA100 hospital background revealed genomic features that may have contributed to its persistence.

Complete genome analysis of the outbreak clone revealed a pattern of genetic changes that matched patient locations, suggesting that transmission bottlenecks and local environmental contamination led to a unique genetic signature at each site. Some isolates and isolate subgroups were separated by >10 variants, which is relatively high considering a reported core genome mutation rate of 2.7 to 3.3 mutations per Mb per year (14, 54). This suggests that the outbreak may have originated from a genetically heterogeneous source, such as a patient with a history of persistent MRSA colonization that accumulated intrahost variants. It is also possible that the combination of selection pressures and transmission bottlenecks contributed to the diversification of the outbreak clone. Considering all available data, we think the most likely scenario is that the NICU outbreak originated from patient p64 and then spread to other adult patients through direct or indirect contact in shared wards. Ventilator 1, used by adult p64 and infant p150, was the most likely vector for entry into the NICU. Ventilator 4 may have provided a potential second entry route via p151, with subsequent transmissions to p141 and p148 (p151 and p141 had an overlapping stay in the PICU). Such a secondary introduction may explain why the p141 and p148 isolates were more distantly related to all other NICU isolates. We were not able to confirm this scenario, as the isolates from p151 were no longer available. All subsequent cases could be explained by location relative to other MRSA colonized patients or sharing of MRSA-exposed ventilators.

The outbreak strain genome differed from the hospital background by multiple mutations of core genes as well as accessory gene gain and loss. Hundreds of genes were impacted by DNA methylation changes in the gene body or promoter regions, but such genes were depleted rather than enriched among differentially expressed genes. As such, the impact of the methylation changes on the outbreak clone (if any) was unclear. Nonetheless, a common theme among the genetic and expression changes was the relevance of genes involved in biofilm formation, persistence, and quorum sensing. Although the collective impact of the mutations will require further investigation, we speculate that these changes may have contributed an increased persistence of the outbreak clone in the environment.

Complete genome data from our hospital-wide screening program provided key information for outbreak management and investigation that could not have been obtained by molecular typing. First, it provided conclusive differentiation of outbreak from nonoutbreak isolates within 10 to 14 days while the outbreak was ongoing, which helped delineate the final case set, identify transmission events, and determine when the outbreak ended. Second, a retrospective analysis of all genetic differences between outbreak cases allowed us to identify subtransmissions and better understand the chain of events that led to each subtransmission. Third, a retrospective analysis integrating all hospital-wide genomic surveillance data indicated that the NICU outbreak had originated much earlier in unrelated adult wards in a different building and, together with electronic location tracking data, helped identify ventilators as likely transmission vectors.

There are some limitations to our study. Our genomic survey was limited to first positive single-patient bacteremias, and additional transmissions may have been missed by excluding nonblood isolates. Moreover, by sequencing single colony isolates, we likely did not fully capture intrahost heterogeneity. Although such heterogeneity may be less common among bacteremias, we did encounter variation within some patients, which was considered when establishing our transmission thresholds. Finally, while we believe that we reconstructed the most likely transmission routes and vectors, we could not definitively link ventilators to the outbreak because we did not detect MRSA contamination. This may be explained by the fact that some ventilators were no longer available for testing when environmental surveillance was initiated, while others were only tested after the transmission events they were implicated in. Nonetheless, it is possible that other factors such as spread by HCWs and/or other vectors contributed as well.

In conclusion, we find that the application of routine genome sequencing in the clinical space provides significant benefits for infection prevention and control. In addition to providing contemporary data on the genomic characteristics of circulating lineages, timely directed intervention and containment of identified transmission events can help prevent further outbreak progression. Although our screening program was limited in scope to bacteremias, early detection of a transmission event between the adult and NICU ward could conceivably have allowed staff to intervene earlier. Accumulating a larger repository of complete and unique genome references and variants associated with successful spreading strains may be key to future outbreak detection and prevention programs by providing high-resolution feature sets for prospective and retrospective data mining purposes.

## Supplementary Material

Supplemental file 1

Supplemental file 2

Supplemental file 3
